# Magnesium Promotes the Regeneration of the Peripheral Nerve

**DOI:** 10.3389/fcell.2021.717854

**Published:** 2021-08-11

**Authors:** Jingxin Zhang, Binjing Zhang, Jinglan Zhang, Weimin Lin, Shiwen Zhang

**Affiliations:** ^1^State Key Laboratory of Oral Diseases & National Clinical Research Center for Oral Diseases, West China Hospital of Stomatology, Sichuan University, Chengdu, China; ^2^Department of Oral Implantology, West China Hospital of Stomatology, Sichuan University, Chengdu, China

**Keywords:** peripheral nerve repair, nerve regeneration, magnesium, biodegradability, biocompatibility

## Abstract

Peripheral nerve injury is a common complication in trauma, and regeneration and function recovery are clinical challenges. It is indispensable to find a suitable material to promote peripheral nerve regeneration due to the limited capacity of peripheral nerve regeneration, which is not an easy task to design a material with good biocompatibility, appropriate degradability. Magnesium has captured increasing attention during the past years as suitable materials. However, there are little types of research on magnesium promoting peripheral nerve regeneration. In this review, we conclude the possible mechanism of magnesium ion promoting peripheral nerve regeneration and the properties and application of different kinds of magnesium-based biomaterials, such as magnesium filaments, magnesium alloys, and others, in which we found some shortcomings and challenges. So, magnesium can promote peripheral nerve regeneration with both challenge and potential.

## Magnesium and Peripheral Nerve Injury

Magnesium is the fourth abundant mineral ion in the human body. Magnesium is involved in more than 300 kinds of enzymatic reactions (Alawi et al., 2018) and various metabolic cycles, playing a significant role in cellular energy metabolism, synthesis of nucleic acid, protein, and cytokine, regulation of various transporters and ion channels, and plasma membrane integrity (Romani, 2011; de Baaij et al., 2015). Therefore, it is necessary to take magnesium regularly for preventing magnesium deficiency. Studies have shown that magnesium deficiency can induce inflammatory syndrome, which is characterised by macrophage activation and release of inflammatory cytokines and acute-phase proteins (Pan et al., 2011), closely related to a variety of chronic and inflammatory diseases such as asthma, attention deficit hyperactivity disorder, diabetes mellitus, cardiovascular disease, migraine headaches, and osteoporosis (Song et al., 2005; Gröber et al., 2015; Dominguez et al., 2020). What is more, magnesium is also essential for the survival and function of neurons: magnesium is involved in the formation of membrane phospholipids, signal transduction (Stangherlin and O’Neill, 2018), the formation of the myelin sheath (Seyama et al., 2018) and synapse (Sun et al., 2016), and regulating the transmission of neurotransmitters such as dopamine and serotonin (5-HT). Many studies have shown that magnesium can promote axons growth and neural stem cells proliferation (Vennemeyer et al., 2014; Sun et al., 2016), regulate the inflammatory response, and then inhibit apoptosis. Magnesium deficiency is closely related to diabetic peripheral neuropathy (DPN) (Dong et al., 2011; Hyassat et al., 2014; Chu et al., 2016; Zhang et al., 2018) and various neurodegenerative diseases such as Parkinson’s disease and Alzheimer’s disease (Oyanagi et al., 2006; Yamanaka et al., 2019). Therefore, magnesium plays an essential role in maintaining the health of the nervous system (Botturi et al., 2020).

Peripheral nerve injuries are usually caused by inflammation, tumours, and traumatic injuries, accounting for 3–10% systemic trauma (Lee et al., 2010). There are more than 300,000 cases of peripheral nerve injury worldwide every year (Siemionow and Brzezicki, 2009). Severe peripheral nerve injury can lead to lifelong dysfunction mediated by the injured nerve, high disability rate and cost, which greatly impact individuals and society. After peripheral nerve injury, the injured neurons undergo secondary oxidative stress response and degeneration, leading to the production of inflammatory cytokines such as IL-1, IL-6, TNF-α, and IFN-β, which promote the Wallerian degeneration and apoptosis of Schwann cells (SCs) (Pan et al., 2011). After that, degeneration occurs in the axons and myelin sheath at the distal stump of the injured nerve, causing secondary nerve damage, thus inhibiting the regeneration of the injured nerve and suppressing the recovery of nerve function (Pan et al., 2011; Allodi et al., 2012). After peripheral nerve injury, macrophages and SCs clear the remaining axons and myelin fragments. The proliferated SCs arrange into bundles surrounded by the basement membrane and secret a series of neurotrophic factors and cell adhesion molecules (Belanger et al., 2016). Thus, the axons of the proximal end grow to the distal end of the injury. Unfortunately, in most peripheral nerve injuries, the broken ends are far apart, and the long recovery time can easily lead to irreversible degenerative lesions in the innervated muscles. Therefore, it is almost impossible to achieve complete recovery after peripheral nerve injury without external intervention to guide and accelerate axonal regeneration (Pfister et al., 2011).

At present, many strategies have been adopted for peripheral nerve injury repair, and autografts are considered to be the “gold standard” clinically. At the same time, there are some insurmountable limitations of autologous nerve graft, such as the risk of donor site lesion, the limited supply and limited therapeutic effect, the risk of pathological changes in the donor site, the limited source of donor nerve and the limited therapeutic effect (Vennemeyer et al., 2015). To replace autografts, nerve conduits made of biomaterials have become a new choice. An ideal nerve conduit should provide mechanical guidance and a suitable microenvironment for nerve axon regeneration and also protect the regenerated nerve. The materials used for nerve conduits mainly include extracellular matrix, natural polysaccharides/proteins, and synthetic polymers (Fei et al., 2017). Studies have shown that they have the same effect as autografts on peripheral nerve repair (Jang et al., 2016). However, due to the lack of mechanical, their ability to repair long gap injuries beyond 20 mm is limited. Therefore, there is an urgent need for advanced materials to reach an ideal nerve repair. Based on the protective effect of magnesium on the nervous system, magnesium begins to be used in the treatment of peripheral nerve injury in recent years, and related research has developed rapidly. In this review, we are aiming at providing an overview of magnesium in promoting peripheral nerve regeneration.

## The Effect and Mechanism of Magnesium ion on Peripheral Nerve Regeneration

Magnesium ion is one of the necessary ions for cell metabolism. It is abundant in cells and participates in hundreds of enzyme reactions in the body. In the local microenvironment, magnesium ion has been proved to regulate mitochondrial calcium buffering as the second messenger, also with the effects of anti-apoptosis, anti-oxidation and anti-inflammatory, which is also of great significance for nerve impulse conduction. At present, most of the studies on the nervous system of magnesium ion focus on craniocerebral injury and spinal cord injury (Vennemeyer et al., 2014; Li W. et al., 2015; Jia et al., 2016), while only a few studies focused on peripheral nerve injury. According to the existing studies, the potential mechanism of magnesium ion in peripheral nerve injury repair can be summarised as the following aspects:

### Nerve Protection

The protective effect of magnesium ions on the central nervous system has been studied. Clinically, magnesium sulfate has been used to prevent cerebral palsy in preterm infants. Wolf et al. (2020) has revealed that in the case of imminent preterm birth, prenatal treatment with magnesium sulfate during 24–32 weeks of gestation reduced the risk of moderate to severe cerebral palsy and had a neuroprotective effect. In addition, magnesium has been suggested to improve functional neurological outcomes in patients with global cerebral ischemia associated with cardiac surgery and cardiac arrest (Pearce et al., 2017). Identically magnesium also exerts neuroprotective effects on peripheral nerves.

It is generally believed that magnesium ions can play a neuroprotective role by inhibiting the secondary injury after nerve injury through regulating cell function, antagonizing of N-methyl-D-aspartic acid receptor (NMDA receptor) and calcium ions (Koltka et al., 2011). In addition to the degeneration of the damaged nerve stump, the uninjured nerve might also suffer from degeneration. Under the stress state, the magnesium ion level decreases. Then the calcium ion channel opens massively, and the calcium ion flows inside, leading to cell swelling and apoptosis. Thus, a supplementary magnesium ion can block the ion channel of NMDA receptor through the action of electric charge, thereby inhibiting the entry of calcium ion into cells and antagonizing the changes in cell permeability caused by injury and the neurotoxic effect of calcium ion (Jia et al., 2016).

Lambuk et al. (2017) found that the excitatory toxicity of glutamate plays a crucial role in the loss of retinal ganglion cells (RGCs) in glaucoma. The toxic effect of glutamate on RGCs was mediated by overstimulation of NMDA receptors. At the same time, intravitreal injection of magnesium Acetyltaurate (MgAT) could prevent NMDA-induced retinal and optic nerve damage (Lambuk et al., 2017).

### Inhibition of Inflammatory Response

Inhibition of the inflammatory response promotes nerve regeneration by preventing SCs from apoptosis (Pan et al., 2009a,b,c), and magnesium supplements have also been reported to prevent motor neuron death in neonatal sciatic nerve injury (Nikolaos et al., 2004). Magnesium ion can promote peripheral nerve repair by inhibiting inflammation.

Pan et al. (2011) found that a high magnesium diet significantly increased magnesium concentrations in plasma and nerve tissue, which improved neurobehavioural and electrophysiological functions and reduced the deposition of inflammatory cells (such as macrophages) and inflammatory cytokines expression. High magnesium supplementation inhibits the expression of MCP-1 and RANES, thereby inhibiting macrophage deposition and myelin clearance (Pan et al., 2009c). Meanwhile, macrophages are a principal source of many inflammatory factors. However, it should be noted that the low magnesium environment might exert an opposite effect of enhancing inflammation in the damaged nerves compared with high concentration. Meanwhile, Pan et al. (2011) also found that high magnesium supplementation could reduce the apoptosis of SCs by promoting the expression of bcl-2 and bcl-X_*L*_ and then down-regulating active caspase-3 and cytochrome expression.

Plenty of evidence demonstrates that magnesium ion is closely related to inflammation. Magnesium has been shown to inhibit activated macrophage-induced inflammation and thus promote the differentiation of mesenchymal stem cells (Hu et al., 2018). In addition, magnesium deficiency leads to significant increases in pro-inflammatory cytokines such as tumour necrosis factor-α, IL-1, and IL-6 (Nielsen, 2018). Arfuzir et al. (2020) observed that pre-treatment with MgAT prevented endothelin-1-induced IL-1β, IL-6, and tumour necrosis factor-α(TNF alpha) expression increasing, and also protected rats from endothelin-1-induced activation of nuclear factor κB (NF-κB) and c-Jun.

However, the underlying mechanisms of magnesium deficiency causing inflammatory response are not fully understood. Possible mechanisms include: (1) Cell entry of calcium and initiation of phagocytes; (2) Calcium channel opening and NMDA receptor activation; (3) Release of neurotransmitters such as substance P; (4) Membrane oxidation and activation of NF-κB.

### Promote Schwann Cell Proliferation and Axon Regeneration

Schwann cells are neuro-gliocytes that constitute the peripheral nervous system. It not only participates in the formation of the myelin sheath but also maintains the growth environment of the axons through its rapid proliferation, division and secretion of various protein molecules after peripheral nerve injury and also promotes the self-repair and regeneration of nerves.

After peripheral nerve injury, SCs secrete large amounts of endogenous neurotrophic factor (NTF), including nerve growth factor (Guo et al., 2018). NGF plays a promoting role in the repair and regeneration of injured peripheral nerves (Shen et al., 2020), which can induce the growth of neuronal processes, prevent neuron degeneration and apoptosis, and then promote the regeneration and repair of injured nerves. It has been reported that NGF acts on the growth of axon fibres rather than neurons themselves (Kumamoto et al., 2015), which proves that it is feasible to apply topical drugs to promote the regeneration of the nerve.

Appropriate concentrations of magnesium ions in the tissue microenvironment can promote SCs proliferation (Monfared et al., 2018a), as well as SCs synthesis and NGF secretion. Therefore, magnesium filaments play a role in promoting the regeneration of neuronal axons, which is manifested by an increase in the number of regenerated axons and the cross-section of regenerated myelinated nerve fibres (Li B. H. et al., 2016). However, the effect of magnesium ions on the promotion of SCs proliferation is not positively correlated with its concentration. Excessive magnesium ion concentration had been reported to inhibit SCs proliferation (Monfared et al., 2018a).

## The Corrosion of Magnesium

The ideal degradation rate of magnesium should match the growth rate of nerve tissue, but in reality, the degradation rate of magnesium is faster than the growth rate of tissue, which fails to provide long-term physical support and play the biological function of promoting nerve regeneration (Kiani et al., 2020). *In vivo*, magnesium mainly undergoes electrochemical reaction, which is affected by the properties of the material itself and the local microenvironment such as pH and protein. Magnesium mainly undergoes pitting corrosion *in vivo*, which is more likely to lead to material fracture and is not conducive to nerve regeneration (Raman and Harandi, 2017).

The corrosion process of magnesium can be divided into the following two steps: (1) In electrolyte solution, magnesium acts as anode to occur galvanic cell reaction, losing electrons and generating Mg^2+^, while water gets electrons and generates H_2_ and OH^–^. Then Mg^2+^ reacts with OH^–^ and forms Mg(OH)_2_, which is deposited on the surface of the material and has a certain protective effect on magnesium; (2) As time goes on, Mg(OH)_2_ reacts with Cl^–^ in body fluid to form MgCl_2_ which is more soluble in water, resulting in Mg(OH)_2_ falling off and magnesium corrosion. In order to have a better application in magnesium promoting peripheral nerve regeneration, the improvement of material properties can provide enough support to maintain sufficient strength and be gradually degraded and absorbed by the human body (Table 1; Williams et al., 2015; Charyeva et al., 2016).

**TABLE 1:** The corrosion resistance improvement of Magnesium.

**Materials**	**Electrolyte**	**Immersion time**	**Corrosion resistance**	**Corrosion characterisation**
				
Mg-10Li	DEME	14 days	↑	pH
	DEME with FBS			
ZN20	DEME	14 days	↑	pH
	DEME with FBS			
NZ20	DEME	14 days	↑	pH
	DEME with FBS			
Mg-Zn-Ca metallic glass system	DMEM/F12	7 days	↑	(1) i_*corr*_(A/cm^2^):crystallised ribbon:7.94 × 10^–6^/relaxed ribbon:5.24 × 10^–6^
				(2) E_*corr*_(V/SCE):crystallised ribbon:-1.38/relaxed ribbon: -1.21
PEDOT on Mg	SBF	-	↑	i_*corr*_(A/cm^2^):Mg:9.08 × 10^–4^/coated Mg:6.14 × 10^–5^
PEDOT/GO on Mg	PBS	22 days	↑	(1) Magnesium ion release reduce 41.1%
				(2) OH^–^ production decrease 43%
				(3) i_*corr*_(A/cm^2^):Mg:49.6 × 10^–4^/coated Mg:12.3 × 10^–5^
				(4) E_*corr*_(V/SCE):Mg:-1.705/coated Mg:-1.55
TA/PVPON on Mg_70_Zn_26_Ca_4_	PBS	14 days	↑	(1) Magnesium ion release reduce
				(2) i_*corr*_(A/cm^2^):Mg_70_Zn_26_Ca_4_:3.89 × 10^–4^ 2 layers:3.24 × 10^–7^ 5 layers:2.75 × 10^–7^ 10 layers:3.16 × 10^–7^
				(3) E_*corr*_(V/SCE):Mg_70_Zn_26_Ca_4_:-1.22 2 layers:-0.26 5 layers:-0.20 10 layers:-0.22
HA on WE43	12 weeks (*in vivo*)		↑	(1) maintain a well-integrated structure
				(2) no gas formation
PGSM-Mg	PBS	28 days	↑	(1) a mass loss of 22.5 ± 3.3%
				(2) Magnesium ion release 54.8 ± 4.2%
				(3) pH stable
PHBV with MgOl	FBS	15 days	↑	inhibit water inter-action and erosion

*“−” means it did not be studied. NZ20: Mg-Sn-Zn; ZN20: Mg-2Zn-Nd; PEDOT: Poly (3,4-ethylenedioxythiophene); GO: graphene oxide; TA/PVPON: tannic acid/poly (N-vinylpyrrolidone); HA: hydroxyapatite; WE43: Mg, 3.78%Y,2.13%Nd,0.46% Zr; PGSM: poly(glycerol-sebacate-maleate); PHBV: poly (3-hyroxybutyric acid-co-3-hydroxyva-leric acid); MgOl: magnesium-oleate; DEME: Dulbecco’s Modified Eagle Medium; FBS: 10 vol% foetal bovine serum; aCSF: artificial cerebrospinal fluid; PBS: phosphate buffer solution; SBF: simulated body fluid.*

## Magnesium in Promoting Peripheral Nerve Regeneration

### Magnesium Metal

The structural and functional damage of the peripheral nerve could be caused by various factors. The current “gold standard” of clinical treatment of peripheral nerve repair replaces the injury gap with an autograft. However, given the limitations of autografts, including the risk of donor site lesion, the limited supply and therapeutic effect, nerve guide conduit has gradually become a more potential alternative (Vijayavenkataraman, 2020). However, due to the lack of mechanical support provided by the empty conduits for nerve regeneration, the empty conduits fail to induce satisfying axon regeneration when injury gaps are more than 1 cm in length (Kuffler and Foy, 2020). Therefore, magnesium metal with excellent biocompatibility and degradability can be used as scaffold material in nerve conduit to provide mechanical support and promote nerve regeneration.

Li B. H. et al. (2016) bridged the two ends of the injured nerve for acute sciatic nerve compression injury model with magnesium wire. The results demonstrated that sciatic functional index (SFI), NGF, p75 neurotrophin receptor and tyrosine receptor kinase A expression increased in the magnesium group after 4 weeks, which suggested that magnesium wire could provide linear support for nerve regeneration thus improving peripheral nerve regeneration across the injury gap. However, the repair effect varies with the different lengths of the injury gap. Hopkins et al. (2017) compared the effect of magnesium filaments used in short and long gaps. The results suggested that regenerating nerve cells and axons could be seen at the end of the nerve stumps in the short gap repair; While in the long gap repair, although functional improvement in the isografts group was not observed in empty conduits plus magnesium wire group, it still found that regenerating tissue associated with the nerve stumps, which proved that magnesium wire could improve histological indexes of regenerated tissues.

Besides, the degradation rate of magnesium filament will also be affected by the filler in the conduit. Vennemeyer et al. (2015) repaired a 6 mm gap of the adult rat sciatic nerve with magnesium filaments placed in poly(caprolactone) nerve conduits for 6 weeks. The results showed that magnesium filaments absorbed actively were surrounded by regenerating nerve tissue, and inflammation caused by magnesium implants was mild. However, there is no synergistic effect between magnesium wire and keratin, which probably was related to the accelerated corrosion of magnesium filaments caused by keratin hydrogel.

In conclusion, magnesium metal is widely used for peripheral nerve repair due to the following advantages: (i) Magnesium metal is featured by rapid and complete degradation; (ii) Magnesium metal has good histocompatibility. While other biomaterials may lead to severe inflammation during absorption, magnesium metal has less tissue stimulation and does less damage to the regenerating nerve tissue; (iii) During degradation, soluble magnesium ions and hydrogen are released, which have neuroprotective and antioxidant effects; (iv) Compared with other metal materials, magnesium metal has better flexibility and smoother surface, which are conducive to the adhesion of SCs; (v) The electrical conductivity of magnesium metal may provide electrical stimulation path for the injured nerve and its dominated muscle, thus contributing to nerve regeneration (Gordon, 2016).

However, magnesium metal still faces many challenges in clinical application: (i) Magnesium metal is not strong enough or degrades too fast to provide enough mechanical support for nerve regeneration during the whole healing process; (ii) The rapid degradation of magnesium metal results in the accumulation of degradation products and then does harms to tissues. Soluble magnesium ions and hydrogen released will increase pH, thus interfering with nerve generation; (iii) Compressive stress can also affect the degradation rate of magnesium metal and even lead to the fracture of magnesium metal. Therefore, faced with these problems, the magnesium metal to be used in clinical treatment should be improved further to slow degradation rate, which can be achieved by alloying with other metals, altering the surface by anodization or physical treatment and coating with water-resistance substances like polymers (Bordbar-Khiabani et al., 2019).

### Magnesium Alloy

As a new biodegradable bimetallic material, magnesium alloy has been widely studied and has a certain potential in applying nerve guidance conduit (NGC). Compared with pure magnesium, magnesium alloy has the following characteristics: (1) In addition to magnesium ion, other metal ions such as Li, Zn, and Ca can be released to facilitate nerve repair (Fei et al., 2017); (2) The degradation rate is more likely to adjust than pure magnesium; (3) Zinc, aluminium, manganese, calcium, lithium, zirconium, rare earth, and other elements are commonly used in magnesium alloys. The corrosion properties, mechanical properties and biocompatibility of magnesium alloys may can be adjusted by changing the composition of elements (Li X. et al., 2016). At the same time, degradation rate, mechanical properties and cytotoxicity may vary with the changing composition of the alloy.

Fei et al. (2017) chose three magnesium alloys with good mechanical alloys with good mechanical properties, namely NZ20 (Mg-Sn-Zn), ZN20 (Mg-2Zn-Nd), and Mg-10Li, to explore their performance. The degradation was found to be: ZN20 < NZ20 < Mg-10Li, and researchers found the potential and feasibility of Mg-10Li and ZN20 in nerve repair, and NZ20 has potential cytotoxicity. However, the experiment has several limitations. Instead of using SCs, which are involved in peripheral nerve regeneration, the researchers selected endothelial cells. At the same time, the experiment was limited to the short term, and the long-term effects of magnesium alloy on neurons are still unclear. Given the limited existing results, more experiments are still needed to explore the impact of composition of magnesium alloy. At the same time, the degradation rate of magnesium alloy and the release rate of magnesium ions still cannot meet the needs of long gap nerve repair. On the basis of this experiment, magnesium alloy shows noticeable potential, and may be promising in promoting regeneration of peripheral nerve. Potential improvement directions include (1) Surface modification, such as surface coating, ion implantation, laser surface melting, and surface nano-crystallization. (2) Structure design: phase structure, particle structure, and amorphous structure (Li X. et al., 2016).

### Mg-Based Metallic Glasses

Metallic glasses, also known as an amorphous alloy or liquid metal, have attracted significant attention. The amorphous structure imparts higher strength, higher wear resistance and corrosion resistance, and good fatigue performance. Magnesium, calcium, zinc, and strontium-based glasses represent a class of biodegradable amorphous alloys. As for magnesium-based glass, many metallic glass systems have been successfully developed, among which the Mg-Zn-Ca metallic glass system possesses a high glass-forming ability and contains the necessary elements of Ca and Zn for the human body (Sun et al., 2012; Dambatta et al., 2015). At the same time, rapid degradation of magnesium is a challenge in long nerve repair. Long nerve distances are difficult to be fully regenerated. It is vital to reduce the corrosion rate and maintain integrity during regeneration. Metallic glass can be a potential substitute for crystalline alloys in neural tissue applications.

Monfared et al. (2018b) used a Mg-Zn-Ca metallic glass system prepared by melt spinning method to prepare relaxed alloys (magnesium-based glass) and crystallised alloys (ordinary alloys), with the main composition of Mg_70_Zn_26_Ca_4_. By impregnation method and potential polarisation method, the result indicates that the relaxed ribbon had higher corrosion resistance. Biocompatibility was studied by MTT assay. The survival rates of the relaxed ribbons and crystallised ribbons were above 75% after 1, 3, and 7 days, indicating their good biocompatibility with the potential for nerve regeneration. However, the cell viability of the relaxed ribbons was higher than that of the crystallised ribbons at all time points, which could be attributed to the fact that the crystallised ribbons had more degradation rate than the relaxed bands. Because the concentration of ions released by the glass system exceeds the optimal concentration, the crystal ribbon released more ions into the medium and exerted toxic effects on cells. The results showed that the corrosion resistance of the relaxed band was better than that of the crystalline band, and it had good cell compatibility with SCs.

Many experiments have demonstrated that the promoting effect of magnesium ion on peripheral nerve regeneration is not positively correlated with the concentration, but there is an optimal concentration. The release rate of magnesium ions in the magnesium-based glass system prepared in this experiment is still beyond the optimal concentration, suggesting that further improving the preparation process and optimising the performance will be the direction of future research.

### Surface Modification of Magnesium and Its Alloys

Although magnesium alloy and Mg-based metallic glass can significantly improve the corrosion resistance of magnesium and slow down the degradation rate, the long-term neurotoxicity of zinc, calcium, and other ions released from magnesium alloys *in vivo* still needs to be considered, and its corrosion resistance also needs to be further improved. Therefore, the surface modification of magnesium and its alloys become the focus of research and application of peripheral nerve regeneration now.

Surface modification methods of magnesium and its alloys mainly includes conversion coating, anodization, micro-arc oxidation and others. In contrast, in terms of biomedical and neural application, the frequently used way of surface modification of magnesium and its alloys is polymer coating by electrospinning, electrochemical deposition, dip-coating, spray-coating, spin-coating, and other ways. Moreover, it will lead to differences in the coating performance if different coating ways are applied (Tian and Liu, 2015).

Poly (3,4-ethylenedioxythiophene) (PEDOT) is an ideal coating material with excellent conductivity and biocompatibility, which has been used in nerve implantation (Richardson-Burns et al., 2007; Krukiewicz et al., 2019). Many types of research have been performed on how the coating improves the properties. Sebaa et al. (2013) modified the Mg surface with PEDOT coating and explored its properties. The adhesion strength of the PEDOT coating was good, in the range of 3–4B measured by ASTM-D 3359 standard tape test. Moreover, the corrosion resistance enhanced with the PEDOT coating. Catt et al. (2017) found that a composite coating composed of PEDOT and graphene oxide (GO) (PEDOT/GO) could significantly enhance the corrosion resistance of Mg. PEDOT/GO coated Mg exhibited lower toxicity and higher biocompatibility. However, Sebaa et al. (2013) lacked the test on the biocompatibility of the PEDOT coated Mg, and they only inferred the role in promoting the peripheral nerve regeneration from the properties of PEDOT instead of measuring the role of the PEDOT coated Mg. Moreover, when Catt et al. (2017) carried out the cytotoxicity experiments, the neurons were cultured only lasted for one day, and the long-term toxicity of the coated Mg has not been clarified.

There are some other coatings to be studied. Monfared et al. (2018a) coated Mg_70_Zn_26_Ca_4_ ribbons (a Mg-based metallic glass) with tannic acid (TA)/poly(N-vinylpyrrolidone) (PVPON). They investigated the corrosion resistance, and biocompatibility was higher than the uncoated and significantly higher than pure Mg. What is more, SCs had an excellent initial attachment with typical morphology on the surface of coated ribbons *in vitro*.

Those studies did not evaluate its promotion effect *in vivo*. Hydroxyapatite (HA) coated magnesium alloy (WE43) *in vitro* improved the adhesion and proliferation of adrenal pheochromocytoma cells (PC12). However, those of SCs were not significantly improved, while *in vivo*, it showed controlled degradation and reduced gas production, but only a little regeneration of sciatic nerve tissue (Almansoori et al., 2018). This research is more convincing to evaluate the properties of HA-WE43 because of the experiments *in vivo*. However, the role in promoting peripheral nerve regeneration is still not good enough, which need to be further improved.

Through most of the above experiments, there was a lack of tests on whether the coated magnesium and its alloys could promote peripheral nerve regeneration, which indicates that it is not rigorous to infer the role of samples after coating based solely on the effect of the coating itself on the peripheral nerve regeneration. Moreover, most of the experiments have only been performed *in vitro* and lacked *in vivo* experiments. What is more, in those studies, the methods to study the properties of the coatings are relatively fragmentary and different, so it is also necessary to establish a more systematic and scientific evaluation system for the properties of the coating. Except for the homogeneity of the coating, more universal properties such as thickness, adhesion, electrical conductivity, degradability, and hydrophobicity should also be included. Strong and homogeneous adhesion is the basis for constructing metals with a stable coating and predicting their properties *in vivo* (Sebaa et al., 2013). Good electrical conductivity and suitable degradation rate are the basis for promoting peripheral nerve regeneration. Hydrophobicity plays a role in isolating Cl^–^ in solution from metals for corrosion resistance (Conceicao et al., 2010). These universal properties are extremely crucial for the application of coated samples, while characteristic properties such as self-healing, negatively charged, and mediating the attachment of another kind of coating are also excellent ways to improve coatings. Self-healing means it can repair its surface coatings and extend the life of the coatings (Fan et al., 2015). Negatively charged itself hinders Cl^–^ in solution from entering the metal surface for corrosion resistance (Prabakar et al., 2013). At the same time, whether the properties of the metals will change after coating also required to be investigated, such as the change of conductivity and the ability to promote peripheral nerve regeneration. Although many challenges remain to be solved, it is undeniable that the coatings of magnesium and its alloy have great potential in promoting peripheral nerve regeneration, such as the combination of coatings with anti-inflammatory drugs to moderate the host response (Zhang et al., 2015), the modification of functional sites of coatings to improve histocompatibility, the combined use of different coatings to improve coating properties (Catt et al., 2017).

### Mg^2+^-Improved Biomaterials

In addition to magnesium wire, magnesium alloy and magnesium and its alloys after surface coating, magnesium ion also possess good neuroprotective and nerve regeneration promoting effect. Thus, the performance of biomaterials improved by incorporation of Mg^2+^ to play its role in promoting peripheral nerve regeneration has attracted many researches.

Sun et al. (2019) hybridised Mg^2+^ and poly(glycerol-sebacate-maleate) (PGSM) into PGSM-Mg and investigated the properties improvement. PGSM-Mg showed suitable biodegradability and sustained release of Mg^2+^. It could promote SCs adhesion and proliferation better and expressed more neural-specific genes than several kinds of scaffolds, showing great potential to promote peripheral nerve regeneration.

Ramburrun et al. (2019) added magnesium-oleate (MgOl) and N-acetyl-L-cysteine (NAC) at different ratios in poly (3-hyroxybutyric acid-co-3-hydroxyva-leric acid) (PHBV) as a different experimental group. The morphology of PC12 was observed as linearly oriented fibre deposition, and cell proliferation was improved. Improved-PHBV had good physical properties, which is favourable for processing and fabricated as a nerve conduit. So, this Mg^2+^-improved biomaterial will hold great promise for applications in promoting peripheral nerve regeneration. If the study explored which concentration of MgOl can better promote SCs growth and proliferation, the study would be more valuable for peripheral nerve regeneration. Moreover, it lacked biodegradability tests, which is also essential for clinical application.

The variousness of peripheral nerve injury makes difficulties regenerating peripheral nerves, so exploring different biomaterials depending on the injury situation and combining with Mg^2+^ will enrich the pathways and fields of promoting peripheral nerve regeneration. Additionally, in order to make Mg^2+^ released at an appropriate rate at the site of the peripheral nerve injury and reduce the toxicity of magnesium, it will be an effective measure to explore how to control the binding and release of Mg^2+^ through biomaterials for the applications of magnesium to promote peripheral nerve regeneration. At the same time, it is necessary to detect and improve the degradability and plasticity of Mg^2+^-improved biomaterials.

## Challenges and Prospects

**FIGURE 1:**
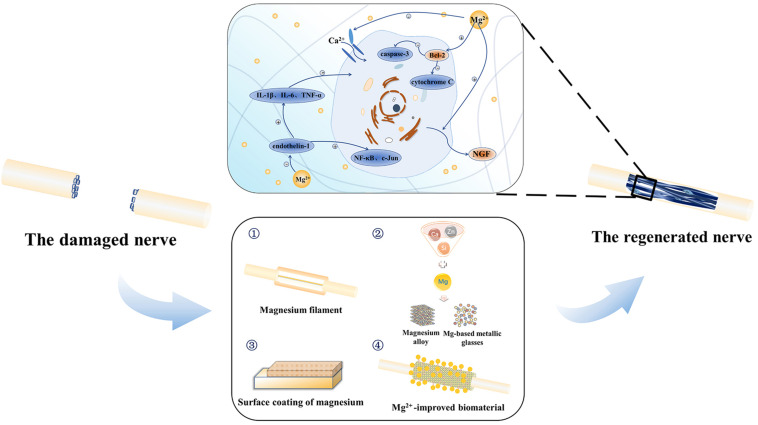
Magnesium promotes the regeneration of the peripheral nerve.

Based on the existing literature, our review not only expounds the importance of magnesium ion to the nervous system and the mechanism of magnesium ion promoting peripheral nerve regeneration but also summarises the application of magnesium-based biomaterials to promote peripheral nerve regeneration by improving the corrosion resistance and biocompatibility (Figure 1 and Table 2). Above all, we can conclude that magnesium can promote peripheral nerve regeneration with both challenge and potential.

**TABLE 2:** Magnesium promotes the regeneration of the peripheral nerve.

**Materials**		**Application**	**Degradability**	**Biocompatibility**	**Regeneration mechanism**	**Future researchimprovement**
Magnesium filaments	Mg	promote nerve regeneration	Yes	Yes	(1) contact guidance	increase the number of animals
					(2) the release of ion may be ineffective	
	Mg	(1) promote nerve regeneration in short gaps	−	Yes	(1) axon nerve regeneration	overcome the problem of a critical gap size
		(2) cannot promote nerve regeneration in long gaps			(2) formation of normal blood vessel	
	Mg, Al, Zn	promote nerve regeneration	−	Yes	(1) promote myelination	explore the degradation of magnesium
					(2) inhibit the inflammation	
Magnesium alloy	Mg-10Li	nerve regeneration potential	ZN20 < NZ20 < Mg-10Li	Yes	Mg-10Li and ZN20 make axon dense and long	(1) reduce cytotoxicity caused by Mg^2+^
	ZN20			Yes		(2) adjust the released amount of other ions
	NZ20			No(cytotoxicity)		
Mg-based metallic glasses	Mg-Zn-Ca metallic glass system	nerve regeneration potential	Yes	Yes	control the corrosion	carry out *in vivo* experiment
Magnesium with surface coating	PEDOT on Mg	(1) nerve regeneration potential	Yes	−	control the corrosion	(1) co-deposition of other coatings
		(2) good adhesion strength of PEDOT				(2) carry out *in vivo* experiment
	PEDOT/GO on Mg	(1) nerve regeneration potential	Yes	Yes	(1) control the corrosion	(1) involve the release of anti-inflammatory drugs
		(2) build-up of negative charges			(2) promote the attachment and neurite extension of primary neuron	(2) provide loci for surfaces functionalization
	TA/PVPON on Mg_70_Zn_26_Ca_4_	(1) nerve regeneration potential	Yes	Yes	(1) control the corrosion	determine the concentration of other ions and their effects on nerve cell
		(2) self-healing ability			(2) promote the attachment of SCs	
	HA on WE43	a little nerve regeneration *in vivo*	Yes	Yes	control the corrosion	improve the flexibility of conduits
Mg^2+^-improved biomaterials	PGSM-Mg	(1) promote SCs adhesion and proliferation *in vitro*	Yes	Yes	enhance the proliferation and neural-specific gene expression of SCs	(1) involve the release of drugs
		(2) sustained release of Mg^2+^				(2) favourable for procession
	PHBV with MgOl	(1) nerve regeneration potential	−	Yes	promote the growth of PC12	(1) carry out *in vivo* experiment
		(2) easily processing				(2) favourable for procession

*“−” means it did not be studied. NZ20: Mg-Sn-Zn; ZN20: Mg-2Zn-Nd; PEDOT: Poly (3,4-ethylenedioxythiophene); GO: graphene oxide; TA/PVPON: tannic acid/poly (N-vinylpyrrolidone); HA: hydroxyapatite; WE43: Mg, 3.78%Y,2.13%Nd,0.46% Zr; PGSM: poly(glycerol-sebacate-maleate); PHBV: poly (3-hyroxybutyric acid-co-3-hydroxyva-leric acid); MgOl: magnesium-oleate.*

(1) The research on the effect and mechanism of magnesium on the peripheral nervous system has not been fully clarified, such as the toxic effect of magnesium. However, its effect and mechanism still play a crucial role in providing effective theoretical guidance for promoting peripheral nerve regeneration.
(2) The research on the properties of modified magnesium needs to be improved. At present, the research mainly focuses on its corrosion resistance and biocompatibility *in vitro*. However, it is still necessary to investigate its role in promoting peripheral nerve regeneration *in vivo* and further confirm the improvement of corrosion resistance and biocompatibility.
(3) The methods to study the properties of magnesium and its alloys are similar and systematic in research, while the methods to study the properties of coatings are relatively fragmentary and immethodical. However, various experimental methods have accumulated rich and valuable experience, which lays a good foundation for improving the coating performance testing system in the future.
(4) Magnesium alloy, its surface coating and other biomaterials modified by magnesium are the main forms to promote peripheral nerve regeneration. So, on the one hand, it is necessary to develop new alloys, coatings or biomaterials, and explore their properties and peripheral nerve promoting effects. On the other hand, in addition to further optimisation on the properties of the alloys, coatings or biomaterials, it is also necessary to consider the cost, efficiency, stability, and other issues, which is expected to play an important role in clinic widely.
(5) The ability of magnesium to promote bone regeneration and vascular regeneration has been widely recognised and has been used in the clinic (Witte, 2010; Chen et al., 2019). Combined with nerve regeneration ability, we can foresee the great potential of magnesium in clinical application, such as dental implants. However, there are still many difficulties to be overcome from the basic to the clinical.

The research on magnesium to promote peripheral nerve regeneration is rising in recent years, but research is relatively deficient. There is still great potential in this field where opportunities and challenges coexist, which needs the joint investment and efforts of many researchers, promoting the development of the research and putting it into clinical use in the future for the benefit of patients!

## Author Contributions

JXZ, BZ, and JLZ summarized the related literature and wrote the manuscript. WL checked and polished the language. SZ contributed to conception, design, and critically revised the manuscript. All authors contributed to the article and approved the submitted version.

## Conflict of Interest

The authors declare that the research was conducted in the absence of any commercial or financial relationships that could be construed as a potential conflict of interest.

## Publisher’s Note

All claims expressed in this article are solely those of the authors and do not necessarily represent those of their affiliated organizations, or those of the publisher, the editors and the reviewers. Any product that may be evaluated in this article, or claim that may be made by its manufacturer, is not guaranteed or endorsed by the publisher.
